# Increased Activity of the Intracardiac Oxytocinergic System in the Development of Postinfarction Heart Failure

**DOI:** 10.1155/2016/3652068

**Published:** 2016-11-10

**Authors:** Agnieszka Wsol, Kaja Kasarello, Marek Kuch, Kamila Gala, Agnieszka Cudnoch-Jedrzejewska

**Affiliations:** ^1^Department of Experimental and Clinical Physiology, Laboratory of Centre for Preclinical Sciences, Medical University of Warsaw, Warsaw, Poland; ^2^Department of Cardiology, Hypertension, and Internal Medicine, 2nd Medical Faculty, Medical University of Warsaw, Warsaw, Poland; ^3^Department of Immunology, Transplantology and Internal Diseases, Transplantation Institute, Medical University of Warsaw, Warsaw, Poland

## Abstract

*Aim.* The present study was designed to test the hypothesis that the development of postinfarction heart failure is associated with a change of activity of the intracardiac oxytocinergic system.* Methods.* Experiments were performed on male Sprague-Dawley rats subjected to myocardial infarction or sham surgery. Four weeks after the surgery, blood samples were collected and the samples of the left ventricle (LV) and right ventricle (RV) were harvested for evaluation of the mRNA expression (RT-PCR) of oxytocin (OT), oxytocin receptor (OTR), natriuretic peptides, and the level of OT and OTR protein (ELISA). The concentration of N-terminal B-type natriuretic peptide was measured to determine the presence of heart failure.* Results.* Plasma NT-proBNP concentration was higher in the infarcted rats. In the infarcted rats, the expression of OT mRNA and the OT protein level were higher in the RV. There were no significant differences between infarcted and noninfarcted rats in the expression of OT mRNA and in the OT protein level in the fragments of the LV. In both the left and the right ventricles, OTR mRNA expression was lower but the level of OTR protein was higher in the infarcted rats.* Conclusions.* In the present study, we indicate that postinfarction heart failure is associated with an increased activity of the intracardiac oxytocinergic system.

## 1. Introduction

For a long time, oxytocin (OT), a neurohypophyseal hormone, was regarded as a pivotal factor in the stimulation of uterine contraction during labour, milk ejection, and the formation of maternal behaviour [1]. In recent years, we have observed how the spectrum of actions of oxytocin has conspicuously widened. Recent advances indicate that OT exerts an antidepressant and anxiolytic effect, and thus it may be a useful agent in the therapy of neuropsychiatric disorders such as autism, depression, social anxiety, and schizophrenia [2–5]. A growing body of evidence has shown that oxytocin plays a multiple role in the central and peripheral regulation of the cardiovascular function. The presence of oxytocin and its receptors was demonstrated in several brain regions involved in the regulation of blood pressure [6, 7]. Several experimental and clinical studies established an OT function in the central and peripheral reduction of arterial blood pressure [8–11]. However, increases in blood pressure following OT central infusion have also been reported in normotensive Wistar-Kyoto rats, but the pressor effect in this case resulted from oxytocin administered in high doses binding to a V1a vasopressin receptor [12].

The mRNA for OT receptors was demonstrated not only in the central nervous system and in the uterus, but also in the atria, ventricles, caval veins, and the aorta. Further observation provided evidence for the heart and vasculature as sites of OT synthesis [13, 14]. In the heart, OT was shown to increase the release of atrial natriuretic peptide (ANP) [15]. Recent studies emphasize the cardioprotective role of OT in experimental models of ischaemic cardiac injury [16–19]. In those studies, OT exerted a cardioprotective mechanism by stimulation of endothelial nitric oxide synthase-guanylate cyclase, ANP-cyclic guanosine monophosphate, phosphoinositide 3-kinase, and protein kinase B pathways and mild activation of mitochondrial ATP-dependent potassium channels (mitoK_ATP_). Activation of the abovementioned processes by OT subsequently resulted in the reduction of the infarct size by the improvement of the following: the cardiac healing process and the remodelling ratio of cardiomyocytes and the interstitial matrix.

The rationale of the present study was to elucidate whether the development of postinfarction heart failure is associated with a change of activity of the oxytocinergic system in cardiomyocytes. Despite promising results of the role of OT in cardioprotection, the activity of the oxytocinergic system in the development of postmyocardial heart failure has not yet been investigated.

## 2. Methods

### 2.1. Animals and Surgical Procedures

#### 2.1.1. Animals

All surgical and experimental protocols were conducted according to the international/EU guidelines and regulations on the use and care of laboratory animals. The experimental protocol was approved by the Second Local Animal Research Ethics Committee. All experiments were performed on 30 male Sprague-Dawley rats (SPRD/Möl/Lod, 250–350 g) bred in the Department of Animal Breeding. The rats were housed (2-3 animals per cage) under standard conditions including 12 h/12 h light/dark cycle (light on at 7.00 a.m.) in a room with regulated temperature (range: 22–25°C) and were fed a standard rat diet and allowed access to water* ad libitum*.

#### 2.1.2. Myocardial Infarction/Sham Surgery

The animals were divided at random into two groups. One group was subjected to myocardial infarction (*n* = 20) and the other group to sham surgery (*n* = 10) at the age of 8–10 weeks. Each of these procedures was performed under pentobarbital anaesthesia (5 mg/100 g body wt. i.p.; 20 *μ*mol/100 g body wt.; Biowet Puławy). At the end of the surgical procedure, the animals were given an analgesic (buprenorphine chloride 3 *μ*g/100 g body wt. i.p., 5.95 nmol/mL, two times daily for 2-3 days) and an antibiotic (penicillin, Polfa 10 000 IU/100 g body wt. i.m.; 0.047 mmol/mL). After the surgical procedure, the rats were placed in separate home cages and remained under observation in the laboratory. After full recovery from anaesthesia, the animals were returned to the animal house.

The myocardial infarction was produced according to the technique described previously [20]. In brief, the rats were subjected to ligation of the left coronary artery just below the exit from the aorta after a surgical incision was made between the IV-V intercostal spaces. In the sham-operated rats, the cardiac pericardium was touched with a needle, but the coronary artery was not ligated. During the surgical procedure, the animals were ventilated by means of air puffs applied with a small rubber balloon. The rate of survival of the infarcted and the sham-operated rats was equal to 50% and 90%, respectively. Of the animals that died, most died within the first 48 hours after surgery. For the molecular and biochemical measurements, tissue was derived from *n* = 10 infarcted and *n* = 9 sham-operated rats.

#### 2.1.3. Tissue Harvesting and Postmortem Examination Procedures

Four weeks after the sham surgery or myocardial infarction, the rats were anaesthetised with pentobarbital sodium (5 mg/100 g body wt. i.p.; 20 *μ*mol/100 g body wt.) and blood samples (2 mL, EDTA) were collected. Then, each rat was sacrificed by decapitation. The heart was excised from the thorax, washed gently with saline, and placed on an iced surface. The ventricles were separated from the atria and the wall of the left ventricle (LV) including the septum was separated from the wall of the right ventricle (RV) along the longitudinal axis. Both ventricles were weighed. The tissue was placed flat on a piece of transparent millimetre paper. The external and internal circumferences of the LV (including the septum) and of the infarcted region were outlined. The surface of the infarct expressed as the number of square millimetres was determined on both sites and averaged. The dimension of the infarct scar was expressed as the percentage of the total left ventricle wall surface as described previously [20–22]. In the previous studies, this method of estimation provided the infarct size consistent with the infarct size obtained by histological examination [20, 22]. For further experiments, the fragments of the left ventricle muscle did not contain the myocardial infarction scar area. All tissue fragments were placed in liquid nitrogen immediately after excision and measuring. To avoid delay in the time of freezing tissues between the infarcted and sham-operated rats, all postmortem experimental processes were conducted by two operators simultaneously. One of the operators performed postmortem procedures on the sham-operated rats, while the other performed them on the infarcted rats. All tissues were frozen almost at the same time. Frozen tissue fragments and plasma were kept in deep freeze at −85°C.

### 2.2. Real-Time Polymerase Chain Reaction (PCR)

Tissue samples were homogenized at a frequency of 25 Hz for 5 minutes in a TissueLyser (Qiagen GmbH, Hilden, Germany) homogenizer. Isolation of total RNA was performed automatically on a BioRobot EZ1 using the EZ1 RNA Universal Tissue Kit (Qiagen, Germany) according to the manufacturer's instructions (Qiagen GmbH, Hilden, Germany). RNA concentration and purity were assessed with a NanoDrop spectrophotometer at 260 nm (ND-1000 Spectrophotometer, Thermo Fisher Scientific Inc.).

Real-time PCR was performed on a ViiA™ 7 Real-Time PCR System using TaqMan® RNA-to-C_T_™ 1-Step Kit (Applied Biosystems, Foster City, USA). Specific primer and probe sets were purchased from Applied Biosystems: oxytocin (Rn00564446_g1), oxytocin receptor (Rn00563503_m1), ANP (Rn00664637_g1), and BNP (Rn00580641_m1) (Table 1). Glyceraldehyde-3-phosphate dehydrogenase (GAPDH; 4352338E) was used as a housekeeping gene. Total reaction volume was 50 *μ*L. The experiment was conducted using MicroAmp™ Optical 96-Well Reaction Plates with Barcode (Applied Biosystems). Amplification was performed in 40 cycles at 95°C for 15 seconds and at 60°C for 1 minute. Duplicates of each sample were performed. The Ct (threshold cycle) for the target gene and the Ct for the internal control were determined for each sample. The relative gene expression was given on the basis of estimations of the values of the delta cycle threshold (ΔCt) by relative quantification to the endogenous control.

### 2.3. Enzyme-Linked Immunosorbent Assay (ELISA)

The following EIA Kits were used for the evaluation of oxytocin, oxytocin receptor concentration in the heart's homogenates, and N-terminal prohormone B-type natriuretic peptide (NT-proBNP) in plasma: Oxytocin EIA Kit (Phoenix Pharmaceuticals Inc., USA), Oxytocin Receptor EIA Kit (Sunred Biological Technology Co. Ltd., China), and NT-proBNP (Wuhan EIAab Science Co. Ltd., China). For the ventricle cardiac muscle, each sample of tissue was homogenized in the TissueLyser bead mixer (Qiagen, USA) and centrifuged (10 000 rpm for 10 minutes, 4°C). The supernatant was collected and frozen at −80°C until analysis. Total protein concentration was measured using bicinchoninic acid (BCA) Protein Assay Kit (Pierce, Holland), according to the manufacturer's instructions. Results obtained were presented as an absolute ratio: concentration/total protein concentration (×10^−9^) for OT and OTR and total protein concentration (pg/mL) for plasma NT-proBNP.

### 2.4. Statistical Analysis

All values presented in the text and figures are means ± SE. STATISTICA software (version 10) was used for statistical analysis of the data. One-way ANOVA followed by the Tukey* post hoc* test was applied for the parameters with normal distribution (OT, OTR, BNP, and ANP mRNA expression, left and right ventricular mass, and body mass). Parameters with nonnormal distribution (OT, OTR protein level, and NT-proBNP plasma concentration) were analyzed with the Kruskal-Wallis test with Dunn's multiple comparison nonparametric test. The differences were considered significant if *P* < 0.05.

## 3. Results and Discussion

### 3.1. Results

Total body mass in the infarcted and sham-operated rats was equal to 287.5 ± 12.1 g and 290.5 ± 6.1 g, respectively, and did not differ significantly. We did not find significant differences between the masses of the left ventricle in infarcted and sham-operated rats (0.22 ± 0.004 versus 0.24 ± 0.01 g). Right ventricle mass was significantly higher in the infarcted rats (0.08 ± 0.004 versus 0.06 ± 0.002 g [*F*(1,17) = 6.417; *P* < 0.05]). The infarct size was 47.3 ± 5.74% of the left ventricle surface.

**Figure 1:**
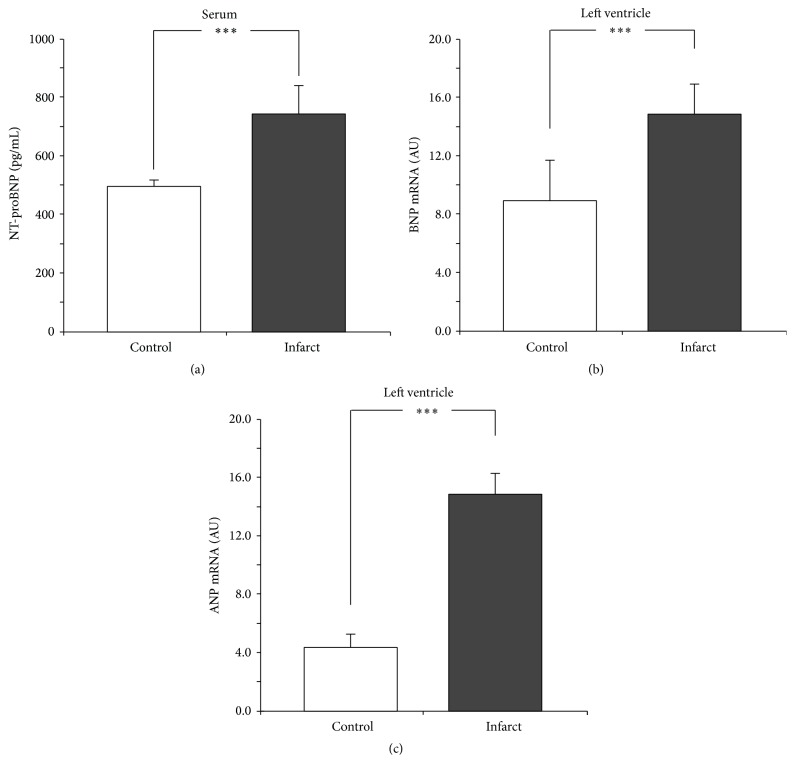
Serum NT-proBNP concentration (a) and average expression of BNP mRNA (b) and ANP mRNA (c) in the fragments of the left ventricle muscle in the sham-operated rats (control) and in rats with myocardial infarction (infarct). Arbitrary units: relative gene expression was given on the basis of estimations of the values of the delta cycle threshold (ΔCt) by relative quantification to the endogenous control. Means ± standard errors are shown. Significant differences between the experimental groups: ^*∗∗∗*^ *P* < 0.001.

Plasma NT-proBNP concentration was significantly higher in the infarcted rats (Figure 1(a)). In the left ventricle, infarcted rats manifested significantly higher expression for BNP mRNA [*F*(1,15) = 428.15; *P* < 0.001] and ANP mRNA [*F*(1,13) = 323.95; *P* < 0.001] (Figures 1(b) and 1(c)).

**Figure 2:**
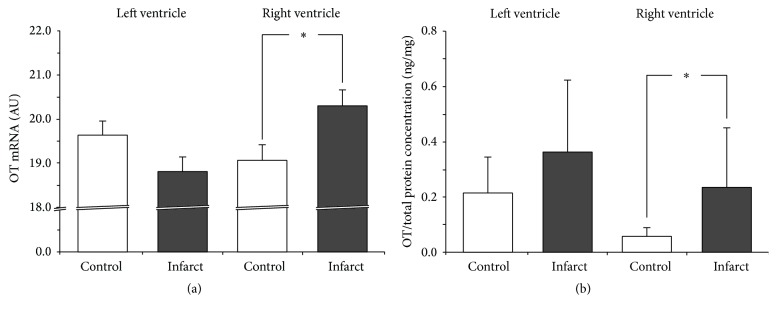
Average expression of oxytocin (OT) mRNA (a) and protein (b) in the left and right ventricle muscles in the sham-operated rats (control) and in rats with myocardial infarction (infarct). Arbitrary units: relative gene expression was given on the basis of estimations of the values of the delta cycle threshold (ΔCt) by relative quantification to the endogenous control. Oxytocin concentration presented as an absolute ratio: oxytocin/total protein concentration. Means ± standard errors are shown. Significant differences between the experimental groups: ^*∗*^ *P* < 0.05.

In the infarcted rats, expression of OT mRNA [*F*(1,14) = 5.699; *P* < 0.05] and OT protein level were higher in the right ventricle (*P* < 0.05). There were no significant differences between infarcted and noninfarcted rats in the expression of OT mRNA and in OT protein in the fragments of the left ventricle (Figures 2(a) and 2(b)).

**Figure 3:**
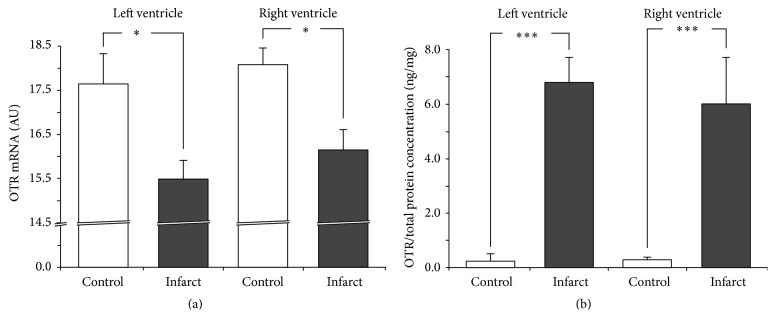
Average expression of oxytocin receptor (OTR) mRNA (a) and protein (b) in the left and right ventricle muscles in the sham-operated rats (control) and in rats with myocardial infarction (infarct). Arbitrary units: relative gene expression was given on the basis of estimations of the values of the delta cycle threshold (ΔCt) by relative quantification to the endogenous control. Oxytocin receptor concentration presented as an absolute ratio: oxytocin/total protein concentration. Means ± standard errors are shown. Significant differences between the experimental groups: ^*∗*^ *P* < 0.05; ^*∗∗∗*^ *P* < 0.001.

We found that the OTR mRNA expression was significantly lower in the infarcted rats both in the left ventricle [*F*(1,12) = 353.75; *P* < 0.001] and in the right ventricle [*F*(1,12) = 73.833; *P* < 0.001], whereas the level of OTR protein was higher in the infarcted rats in both the left and the right ventricles (Figures 3(a) and 3(b)).

### 3.2. Discussion

In the present study, we show for the first time that postinfarction heart failure is associated with significant changes in both the mRNA expression and the protein level of the intracardiac oxytocin and oxytocin receptor. By analyzing the results of protein expression for both OT and OTR, we conclude that the experimental model of postinfarct heart failure was associated with an increase in activity of the heart's oxytocinergic system.

Results of past studies revealed that OT increases the release of natriuretic peptide, a powerful marker of the left ventricle systolic function, from the heart [23–25]. Repeated injections of OT in rats resulted in both an increase of natriuresis and elevation of plasma ANP. Moreover, the stimulation of ANP release by OT was observed also in the absence of neuronal influence in experiments with isolated heart perfusion, which provided evidence for ANP release by the intracardiac oxytocinergic system [15]. In the present study, we show by means of an increase in the expression of ANP, BNP mRNA, and plasma NT-proBNP concentration that our experimental model resulted in the development of heart failure. At the same time, we observed an increase in OT level in the muscle of the right ventricle and an increase in protein synthesis for OTR in both ventricles of the heart. Jankowski et al., in the experimental model of myocardial infarction in rats, reported the reduction of natriuretic peptides by OT infusions as a result of left ventricle systolic function improvement [17]. These results are questionable as, in most of the studies, OT was shown to increase natriuretic peptides (ANP) production [15, 24, 25]. According to the data presented from experimental studies and the results from our observations, we may suggest that an increase in natriuretic peptide in heart failure could result from the increased activity of intracardiac OT. However, further investigations to assess the direct correlation between intracardiac OT activation and natriuretic peptides expression in our experimental model of postinfarct heart failure are needed.

It should be emphasized that in our study we showed, for the first time, changes in the expression of OT and OTR mRNA and the level of OT and OTR protein four weeks after myocardial infarction, which was reported previously as sufficient time for the development of postinfarct heart failure [21, 22]. Most of the up-to-date studies on the role of oxytocin in myocardial infarction were based on experimental methods involving an ischaemia-reperfusion model* in vitro* [19, 26, 27] or* in vivo* [16, 17]. In the study by Jankowski et al., myocardial infarction was evoked by chronic ligation of the left anterior descending coronary artery [17]. In that study, the time of observation was limited only to 7 days after myocardial infarction. Lower OTR mRNA expression observed here is consistent with the results published previously by Jankowski et al. [17]. Nevertheless, the authors of the study reported a reduction in OTR protein in the fragments of the left ventricle, 3 and 7 days after myocardial infarction. The cardiac muscle was not examined in that study for OT content. The increase in OTR protein reported here is observed at a later phase of infarct. This raises an important question of whether the differences between OTR protein level obtained in our study and those in the abovementioned study by Jankowski et al. could result from the changes in OT protein level in tissue samples in the early period after myocardial infarction in comparison with chronic postinfarct heart failure.

In our study, we observed a decrease of OTR mRNA while the OTR protein level in the ventricles of the infarcted rats increased four weeks after myocardial infarction. A suggested mechanism(s) of the observed dissociation between OTR mRNA and its protein may involve desensitization and internalization of OTRs or changes in the regulation of synthesis and degradation of mRNA. Additionally, the dissociation may be disease-dependent, particularly during the progression of heart failure. It has been shown that, in HEK293 cells, internalization of OTR occurs mainly due to the clathrin-dependent pathway, but another mechanism involved in the internalization of OTR into cellular membrane caveolae has been proposed [28]. It is possible that the increased content of OTR observed in our study also involves the population of caveolized OTR inaccessible for oxytocin. Previous studies showed that exposure to oxytocin in human myometrial cells resulted in a 10-fold reduction of oxytocin binding capacity. Moreover, in that experiment, total OTR amount did not change in the first 48 hours after exposure to OT, and OTRs were not internalized in this process. At the same time, OTR mRNA was reduced [29].

In the present study, we found higher expression of OT mRNA and higher protein levels in the fragments of the right but not in the left ventricle. The data from previous studies indicate that the OT concentration is the highest in the right atrium in comparison with the left atrium and in the right ventricle in comparison with the left ventricle cardiomyocytes [13]. This observation suggests that the structures in the “right heart” are more actively synthesizing oxytocin. On the other hand, in our results, we found an increase only in the right ventricle mass, which also signalizes right ventricle hypertrophy, 4 weeks after the left coronary artery ligation. A plethora of evidence showed that stimulation with estrogens increases oxytocin synthesis [30–32]. Activation of estrogen receptors *β* was shown to raise the amount of oxytocin peptide [29]. Moreover, in the study by Cavasin et al., estrogens prevented deterioration of the cardiac function and remodelling in the experimental model of myocardial infarction [33]. Therefore, there is another suggested mechanism that could influence the lack of oxytocin mRNA: protein expression in the left ventricle observed in our study may be correlated with a possible change in estrogen activity (especially via estrogen receptor *β*) in the development of left ventricle remodelling due to myocardial infarction.

The results from the present study may suggest an increased activity of the oxytocinergic system in the heart as another aspect of the OT cardioprotective function in the experimental model of myocardial infarction. Results from recent studies indicate that increased activity of the oxytocinergic system in experimental models of left ventricle damage may slow down the progression of heart failure due to the reduction of the infarct size and cardiac remodelling. In the study by Jankowski et al., the treatment with OT chronic infusions over 7 days following myocardial infarction resulted in the improvement of the left ventricle systolic function, reduction of apoptosis, expression of proinflammatory cytokines transcripts, and inflammatory cell migration in the infarct region [17]. The authors of that study did not measure oxytocin mRNA expression and protein level at the same time; thus, it cannot be excluded that endogenous intracardiac OT could be partially responsible for the observed cardioprotective effects. In the other study, pretreatment with OT before ischaemia-reperfusion exposure resulted in the reduction of infarct size and activation of the prosurvival p38-MAPK and Akt-kinase pathways [26, 27]. In the* in vitro* model of ischaemia-reperfusion-induced myocardial damage, Ondrejcakova et al. showed that exposure to OT prior to ischaemia resulted in the reduction of the infarct size due to the negative chronotropic effect [19].

## 4. Conclusions

In conclusion, the main finding of the present study is that postmyocardial infarction heart failure is associated with an increased activity of the intracardiac oxytocinergic system. As oxytocin was found as a novel and important cardioprotective factor in myocardial ischaemia and was also shown to increase natriuretic peptide release, we therefore suggest that increased activity of the intracardiac oxytocinergic system after left ventricle damage may have an inhibitory impact on the progression of heart failure. According to the importance of this novel issue in the pathophysiology of heart failure, further investigations on oxytocin in heart failure are incontestably necessary.

## Figures and Tables

**Table 1:** Sequences of oligonucleotides used in the present study.

Gene	Sense primer (5′-3′)	Antisense primer (5′-3′)	Accession number
OT, *Oxt*	GACGGTGGATCTCGGACTGAA	CGCCCCTAAAGGTATCATCACAAA	Rn00564446_g1
OTR, *Oxtr*	GTCAATGCGCCCAAGGAAG	GATGCAAACCAATAGACACC	Rn00563503_m1
ANP, *Nppa*	CAGCATGGGCTCCTTCTCCA	GTCAATCCTACCCCCGAAGCAGCT	Rn00664637_g1
BNP, *Nppb*	CCATCGCAGCTGCCTGGCCCATCACT	GACTGCGCCGATCCGGTC	Rn00580641_m1

BNP: B-type natriuretic peptide; OT: oxytocin; OTR: oxytocin receptor.
